# *Aulonastus similis* n. sp., a new quill mite species (Syringophilidae) parasitising passeriform birds (Tyrannidae and Cardinalidae) in Mexico

**DOI:** 10.1007/s11230-016-9653-9

**Published:** 2016-08-13

**Authors:** Lukasz Broda, Miroslawa Dabert, Eliza Glowska

**Affiliations:** 1 Department of Animal Morphology, Faculty of Biology, Adam Mickiewicz University, Umultowska 89, 61-614 Poznan, Poland; 2 Molecular Biology Techniques Laboratory, Faculty of Biology, Adam Mickiewicz University, Umultowska 89, 61-614 Poznan, Poland

## Abstract

A new quill mite species, *Aulonastus similis* n. sp. (Acariformes: Syringophilidae), parasitising *Myiozetetes similis* (Spix) (Tyrannidae) and *Habia fuscicauda* (Cabanis) (Cardinalidae) in Mexico is described and DNA barcode sequences of the mitochondrial cytochrome *c* oxidase subunit I (*cox*1) and D1–D3 region of the nuclear 28S rRNA gene are provided. Morphologically, females of *A. similis* are close to *A. euphagus* Skoracki, Hendricks & Spicer, 2010 but differ from this species in the length ratios of the idiosomal setae: *ve*:*si* (2–2.3:1 *vs* 1:1) and *f2*:*f1* (4.7–6.3:1 *vs* 3.3:1).

## **Introduction**

Mites of the family Syringophilidae (Acariformes: Cheyletoidea) are a highly diverse group of obligatory bird ectoparasites inhabiting the quills of a wide spectrum of hosts (Kethley 1970; Skoracki 2011). Currently, this family is represented by 334 species grouped into 60 genera which were recorded from 482 bird species (belonging to 95 families and 24 orders) (Glowska et al., 2015). The genus *Aulonastus* Kethley, 1970, so far has included 14 species parasitising 17 passeriform species belonging to ten families, Emberizidae (4 species), Motacillidae (3 spp.), Icteridae (2 spp.), Fringillidae (2 spp.), Troglodytidae (1 sp.), Laniidae (1 sp.), Turdidae (1 sp.), Cardinalidae (1 sp.), Prunellidae (1 sp.) and Rhinocryptidae (1 sp.) from Poland, Slovakia, Russia, Chile, Ecuador and the USA (Skoracki et al., 2010; Skoracki, 2011; Glowska et al., 2015).

**Figs. 1–5:**
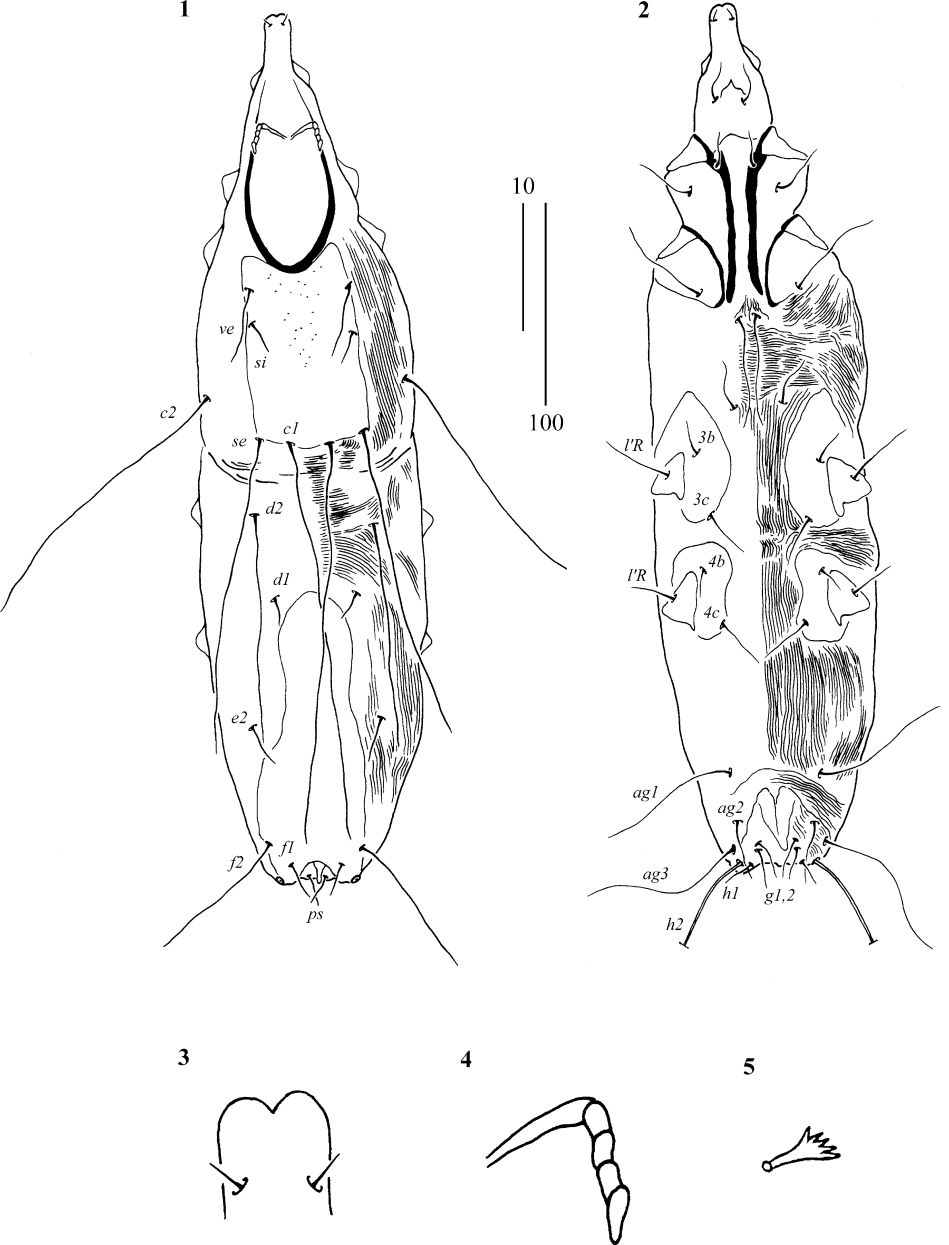
*Aulonastus similis* n. sp., female. 1, Dorsal view; 2, Ventral view; 3, Hypostomal apex; 4, Peritremes; 5, Fan-like setae *p’* of leg IV. *Scale-bars*: 1, 2, 100 µm; 3–5, 10 µm

For a long time, quill mites have been under an extensive taxonomic investigations based entirely on the external morphology (Kethley, 1970; Bochkov & Mironov, 1999; Fain et al., 2000; Skoracki, 2011). However, recent application of molecular tools to syringophilids taxonomy has contributed to both, extending the standard of new species descriptions (Glowska et al., 2012a, b) and verification of compatibility between morphological and molecular delimitations of species boundaries (Glowska et al., 2013, 2014).

In the present paper, a new species of quill mite, *Aulonastus similis* n. sp., parasitising passerine birds of the families Tyrannidae (new host family for the genus) and Cardinalidae in Mexico (a new locality for *Aulonastus* spp.) is described using standard morphological data extended by the generation of novel DNA barcode sequences.

## Materials and methods

*Animal material*

Mites used in the study were collected from the body feathers of the social flycatcher *Myiozetetes similis* (Spix) (Tyrannidae) and the red-throated ant-tanager *Habia fuscicauda* (Cabanis) (Cardinalidae) in Mexico in 2008. Each covert was completely removed from the bird and dissected under an Olympus ZS30 stereomicroscope. Mites were preserved in 96% ethanol and, before mounting on microscopic slides, individually subjected to DNA extraction. Vouchers were mounted on slides in Faure’s medium. Drawings were made with an Olympus BH2 microscope with differential interference contrast (DIC) optics and a camera lucida. All measurements and scale-bars in the figures are given in micrometres. The nomenclature for idiosomal setation is after Grandjean (1939) with modifications adapted for Prostigmata by Kethley (1990), and the nomenclature for leg setation is after Grandjean (1944). The application of these chaetotaxic schemes to Syringophilidae was recently provided by Bochkov et al. (2008) with changes by Skoracki (2011). Latin and common bird names follow Clements et al. (2014). The type-material is deposited in the following depositories: Adam Mickiewicz University, Poznań, Poland (AMU); Museum of Zoology of the University of Michigan, Ann Arbor, USA (UMMZ); Colección Nacional de Ácaros, Instituto de Biología, Universidad Nacional Autónoma de México (UNAM), Mexico (CNAC). The voucher material and the corresponding DNA samples are deposited in the collection of AMU under the identification numbers as indicated below.

*DNA isolation and sequencing*

Total genomic DNA was extracted from single specimens using DNeasy Blood & Tissue Kit (Qiagen GmbH, Hilden, Germany) as described by Dabert et al. (2008). We generated sequences for the mitochondrial cytochrome *c* oxidase subunit 1 (*cox*1) gene and a fragment comprising D1–D3 regions of the nuclear 28S rRNA gene. *Cox*1 was amplified by PCR with degenerate primers: Aseq01F (5′-GGA ACR ATA TAY TTT ATT TTT AGA-3′) and Aseq03R (5′-GGA TCT CCW CCT CCW GAT GGA TT-3′) (Glowska et al., 2014). PCR amplifications were carried out in 10 µl reaction volumes containing 5 µl of Type-it Microsatellite Kit (Qiagen), 0.5 µM of each primer, and 4 µl of DNA template using a thermocycling profile of one cycle of 5 min at 95°C followed by 35 steps of 30 s at 95°C, 1 min at 50°C, 1 min at 72°C, with a final step of 5 min at 72°C. Amplification of 28S rDNA fragments was done with primers 28F0001 (5′-ACC CVC YNA ATT TAA GCA TAT-3′) and 28R0990 (5′-CCT TGG TCC GTG TTT CAA GAC-3′) (Mironov et al., 2012). After amplification, the PCR products were two-fold diluted with water, and 5 µl of the sample was analysed by electrophoresis on a 1.0% agarose gel. Samples containing visible bands were purified with thermosensitive Exonuclease I and FastAP Alkaline Phosphatase (Fermentas, Thermo Scientific). *Cox*1 amplicons were sequenced in one direction using the Aseq01F primer, and 28S rDNA fragments were sequenced in two overlapping fragments using internal primers: sy28SF11 (5′-CAT TTT CAC TCT TCT CAT GC-3′) and sy28SR12 (5′-AGC AAA GCA TAG TAC ACA TTT ATA-3′) (Glowska et al., 2014). Sequencing was performed with BigDye Terminator v3.1 on an ABI Prism 3130XL Analyzer (Applied Biosystems, Foster City, CA, USA). Sequence chromatograms were checked for accuracy and edited using FinchTV 1.3.1 (Geospiza, Inc., Seattle, USA) and manually aligned in GeneDoc v.2.7.000 (Nicholas & Nicholas, 1997). The sequences have been deposited in the GenBank database under accession nos. KU587037–KU587040.

## Results

**Family Syringophilidae Lavoipierre, 1953**

**Subfamily Syringophilinae Lavoipierre, 1953**

**Genus*Aulonastus*Kethley, 1970**

***Aulonastus similis*n. sp.**

*Type-host*: *Myiozetetes similis* (Spix) (Passeriformes: Tyrannidae), social flycatcher.

*Other host*: *Habia fuscicauda* (Cabanis) (Passeriformes: Cardinalidae), red-throated ant-tanager.

*Type-locality*: Los Tuxtlas Tropical Biological Station (UNAM), 18°35′7″N, 95°4′31″W, Veracruz, Mexico.

*Type-material*: Holotype female (UMMZ BMOC 15-0116-1), 13.v.2008, coll. S.V. Mironov (SVM 08-0513-6). Paratypes: three females (AMU-SYR. 1002.1-3, vouchers nos. EG 342, EG343, EG671), two females (UMMZ BMOC 15-0115-1a, b), three females (CNAC), same data as for the holotype.

*Site on host*: Quills of body feathers.

*Voucher material*: Three females from *H. fuscicauda*, collected at Los Tuxtlas Tropical Biological Station (UNAM), 18°35′7″N, 95°4′31″W, Veracruz, Mexico, 1.v.2008, coll. S.V. Mironov (SVM 08-0501-3). All exoskeletons were lost during DNA extraction.

*Representative DNA sequences*. GenBank accession numbers for molecular voucher code EG 342 (ex type-host): KU587039 (*cox1*) and KU587037 (D1–D3); and for EG302 (ex *H. fuscicauda*): KU587040 (*cox*1) and KU587038 (D1–D3).

*Etymology*: The specific name is taken from the specific name of the type-host.

Description (Figs. 1–5)

*Female* [Based on the holotype and 7 paratypes (range in parentheses).] Total body length 410 (380–435). *Gnathosoma.* Infracapitulum apunctate. Each transverse branch of peritremes with 1 long chamber, each longitudinal branch with 4–5 chambers (Fig. 4). Stylophore apunctate, 120 (120–125) long. Movable cheliceral digit (90) long. *Idiosoma.* Propodonotal shield well sclerotised, bearing bases of setae *ve*, *si*, *se* and *c1*, its antero-median part punctate. Length ratio of setae *ve*:*si* 2–2.3:1. Hysteronotal shield fused to pygidial shield, unstriated and apunctate. Length ratios of setae *d2*:*d1* 5.5–7:1, *d1*:*e2* 1:1–2, *f1*:*h1* 1:1–1.2, *f2*:*h2* 1:2.4–3.4, *ag1*:*ag2*:*ag3* 1.6–1.7:1:2.1–2.3. Setae *f2* 4.7–6.3 times longer than *f1*. Setae *h2* 12–16 times longer than *h1*. Genital setae subequal in length. Genital setae *g1*, *2* equal or slightly (1.3 times) longer than pseudanal setae *ps1*. Setae *3c* 1.2 times longer than *3b*. Coxal fields I–IV delicately punctate or apunctate. Cuticular striations as in Figs. 1–2. *Legs*. Fan-like setae *p’* and *p”* with 5 tines (Fig. 5). Setae *tc”* of legs III–IV 1.5 times longer than *tc’*. Lengths of setae: *ve* 20 (right)/30 (left) (35–40); *si* 15 (15–20); *se* (140–155); *c1* 180 (195–220); *c2* (125–145); *d1* 15 (20); *d2* (105–140); *e2* 25 (20); *f1* 15 (15); *f2* 70 (95); *h1* 15 (20); *h2* 240 (215–230); *ps1* 15 (20); *g1*, *2* 20 (15–20); *ag1* 50 (55–80); *ag2* 30 (40); *ag3* 70 (85–105); *l’RIII* 20 (30); *l’RIV* 20 (20); *tc’III–IV* 20; *tc”III–IV* 30; *3b* 15; *3c* 20.

### DNA barcodes

We sequenced 609 bp of the 5′-terminus of the *cox*1 gene and 1,148 bp comprising D1–D3 region of 28S rDNA for three specimens collected from *Myiozetetes similis* and three specimens collected from *Habia fuscicauda*. The *cox*1 sequences revealed two different haplotypes specific for each population. They vary in one nucleotide position leading to no amino acid substitution. The conspecific status of both populations (from *M. similis* and *H. fuscicauda*) is also supported by the 28S rRNA gene fragment because all analyzed specimens showed no variation.

### Remarks

The new species is morphologically most similar to *Aulonastus euphagus* Skoracki, Hendricks & Spicer, 2010, described from the Brewer’s blackbird *Euphagus cyanocephalus* (Wagler) (Passeriformes: Icteridae) in the USA (Skoracki et al., 2010). In females of both species, the transverse and longitudinal branches of the peritremes are represented by 1 and 4–5 chambers, respectively, the propodonotal shield is punctate, the unstriated hysteronotal shield is fused to the pygidial shield, genital setae are subequal in length and coxal fields I–IV are delicately punctate or without punctation. Females of *A. similis* n. sp. differ from *A. euphagus* in the length ratios of setae *ve*:*si* 2–2.3:1 and *f2*:*f1* 4.7–6.3:1. In females of *A. euphagus*, setae *ve* and *si* are subequal in length and the length ratio of setae *f2*:*f1* is 3.3:1.

The presence of *A. similis* n. sp. on two phylogenetically distant hosts belonging to different passerine suborders (Passeri and Tyranni) suggests a distribution resulting from horizontal transfer between two host species rather than cophylogenetic processes.
